# A Serious Game for Performing Task-Oriented Cervical Exercises Among Older Adult Patients With Chronic Neck Pain: Development, Suitability, and Crossover Pilot Study

**DOI:** 10.2196/31404

**Published:** 2022-02-01

**Authors:** Hector Beltran-Alacreu, Gonzalo Navarro-Fernández, Daniela Godia-Lledó, Lucas Graell-Pasarón, Álvaro Ramos-González, Rafael Raya, Aitor Martin-Pintado Zugasti, Josue Fernandez-Carnero

**Affiliations:** 1 Toledo Physiotherapy Research Group Faculty of Physical Therapy and Nursing Universidad de Castilla-La Mancha Toledo Spain; 2 CranioSPain Research Group Centro Superior de Estudios Universitarios La Salle Madrid Spain; 3 Innovation and Research Unit Galeneo Madrid Spain; 4 Physiotherapy Department Centro Superior de Estudios Universitarios La Salle Universidad Autónoma de Madrid Madrid Spain; 5 Departamento de Ingeniería de Sistemas de Información Universidad San Pablo-CEU CEU Universities Madrid Spain; 6 Werium Solutions Madrid Spain; 7 Departamento de Fisioterapia, Facultad de Medicina Universidad San Pablo-CEU CEU Universities Madrid Spain; 8 Department of Physical Therapy, Occupational Therapy, Rehabilitation and Physical Medicine Rey Juan Carlos University Madrid Spain; 9 Grupo Multidisciplinar de Investigación y Tratamiento del Dolor Grupo de Excelencia Investigadora Universidad Rey Juan Carlos-Banco de Santander Madrid Spain

**Keywords:** video games, neck pain, aged, virtual reality, exercise therapy, physical therapy modalities, technology

## Abstract

**Background:**

There is sparse research on the effectiveness of therapeutic exercise for the treatment of neck pain in older adult populations. Moreover, there is a lack of research on the use of serious games or virtual reality for the treatment of neck pain in this population.

**Objective:**

The primary aim of this study was to develop and assess the suitability of a serious game for performing task-oriented cervical exercises in patients with neck pain.

**Methods:**

A serious game was designed based on the key features identified by previous studies that designed serious video games for physical and cognitive rehabilitation or exercise. The game in this study was designed to provide an interactive scenario, with the main functionality of the software solution to control a virtual airplane to reach targets using head motions. At the end of the exercise, the application stores the targets reached and missed and the airplane’s trajectory. A crossover pilot study was carried out for preliminary evaluation of the suitability of the technology in the older adult population. Men and women over 65 years of age with chronic neck pain were included. Subjects were randomly assigned to two study arms; each arm consisted of a sequence of two 4-week treatments with an intermediate washout period of 4 weeks. The total study duration was 16 weeks due to a final follow-up measure 4 weeks after the end of all treatments. Treatment A consisted of the use of the serious game developed in this study, and treatment B consisted of conventional exercises. Subjects allocated to the A-B study arm received treatment A first, followed by treatment B, and vice versa in the B-A arm. The following variables were assessed: Suitability Evaluation Questionnaire (SEQ) scores, Visual Analog Scale scores, and the number of targets reached in the serious game.

**Results:**

A total of 18 subjects were assessed for eligibility. A total of 13 subjects, aged between 71 and 92 years (mean 81.85, SD 6.82), were finally included and completed the study protocol. The global mean SEQ score was 50.38 (SD 5.35) out of 65 points, showing good suitability of the serious game. Most patients considered the experience very enjoyable and “real” in terms of the virtual environment and found the information provided to be clear. Also, they believed that the game could be very helpful for their rehabilitation. None of the patients felt any neck pain or discomfort when playing the game, and only 2 patients out of 13 (15%) reported some degree of dizziness, eye discomfort, or disorientation, which did not limit their capacity to finish the session.

**Conclusions:**

The serious game developed in this study showed good suitability for use in adults over 70 years of age with chronic neck pain. The game was a safe method for performing task-oriented cervical exercises, and patients reported very high levels of satisfaction and acceptance after the use of this technology.

## Introduction

Neck pain is a highly prevalent musculoskeletal disorder among populations of developed societies that leads to considerable pain, disability, and economic burden [1]. The 1-year prevalence of neck pain in the general population has been shown to be 25.8% (range 4.8%-79.5%) on average, with a point prevalence of 14.4% (range 0.4%-41.5%) [2]. It has been ranked as the 4th-greatest contributor to overall global disability and 21st in terms of overall burden [3]. Between 50% and 85% of the general population who experience neck pain at some point in their lives will report it again 1 to 5 years later [4]. Neck pain has been shown to be higher in females and to increase with age, up to 70 to 74 years, then to decrease with older age [1]. However, results from the Spanish National Health Survey showed that the age group of individuals older than 75 years had a higher prevalence of chronic neck pain (17.32% of males and 34.60% of females) compared to other age groups [5].

Clinical guidelines recommend therapeutic exercise as one of the main therapeutic options for patients with neck pain [6]. However, recent systematic reviews have highlighted the need for further research, as there has only been low- or moderate-quality evidence to support its effectiveness [7,8].

The use of technology in the context of the prescription or performance of therapeutic exercise in patients with neck pain has become more popular in recent years. Serious gaming has been described as the use of computer games where the primary goal is not pure entertainment [9]. Serious games have shown positive clinical results in physical rehabilitation [10] and pain management contexts [11]; they are believed to increase motivation and engagement in health care contexts, in which patients are required to undergo repetitive or mundane tasks that can be perceived as boring or nonmotivating. These games involve participation in challenging game environments that can potentially help patients to be more adherent to treatment regimens, as well as focus their attention on an engaging distraction away from aversive symptoms [9,12].

The use of serious games for neck pain has been evaluated in various investigations, normally described in terms of immersive virtual reality (VR) serious games using head-mounted displays or using nonimmersive flat-screen computer games [13-15]. Recent systematic reviews reported that existing evidence of VR effectiveness in patients with chronic neck pain is promising [15] but recommended further focused, high-quality research due to the low-quality evidence available [14].

Previous research studies have developed serious games or VR systems and investigated their use in terms of assessment of neck kinematics [16,17] and exercise prescription for the treatment of patients with neck pain [18,19]; these treatments showed good psychometric properties [17] and effectiveness in neck pain, disability, satisfaction, or cervical motion kinematics [18]. However, to the authors’ knowledge, no previous research has investigated the suitability of the use of similar technologies in the performance of task-oriented neck exercises in older adult populations with chronic neck pain.

There is sparse research on the effectiveness of therapeutic exercise for the treatment of neck pain in older adult populations [8]. Moreover, there is a lack of research on the use of serious games or VR for the treatment of neck pain in this population [14]. Previous research has suggested that VR technology could improve variables such as functional mobility through improving gait quality and resistance in older populations [20]. A recent systematic review also suggested that VR interventions have the potential to improve health outcomes in older adults. However, factors including frailty as well as usability or acceptability of this technology need to be explored in future research in this population [21].

The use of serious video games with motion capture sensors has become popular in physical treatments in the last decade. Some pieces of commercial equipment, mainly Wii or Kinect, have been applied for clinical purposes among older adult people, especially for balance training [22,23] or pain management [24]. Most of these studies demonstrated that functions such as walking, muscle strength, and other motor functions improved. Despite their potential, these kinds of commercial devices have not been designed to monitor and register relevant parameters, such as range of motion (ROM) or movement velocity, which would be useful for quantifying the progress of the intervention. In addition, they are usually designed to train functional movements of the lower and upper limbs.

The video game in this study is controlled by the ENLAZA sensor (Werium Solutions), which consists of an inertial sensor that translates the cervical ROM into mouse pointer displacements. The ENLAZA interface has previously been used for the following purposes: as an input device [25], for physical rehabilitation [26], and for biomechanical assessment [27-29]. These previous studies show the potential of the use of the device in the rehabilitation of cervical movement in people with neurological disorders, such as cerebral palsy. However, this device has never been tested in the population being investigated in this paper.

The primary objective of this study was to develop and assess the suitability of a serious game for performing task-oriented cervical exercises in patients with neck pain. The secondary objective was to compare the effects of the serious game with those of conventional therapeutic exercises among older adult patients with chronic neck pain.

## Methods

### Sensor Description

The sensor development was based on previous work focused on head-mounted interfaces in the field of augmentative and alternative communication for children with cerebral palsy [30,31]. The ENLAZA sensor (Werium Solutions) has also been validated to measure the ROM of different body regions, such as the neck, knee, elbow, and wrist [27-29,32].

The core of the sensor is the MPU-9250 microelectromechanical systems sensor (InvenSense), which integrates a 3D accelerometer, a 3D gyroscope, and a 3D magnetometer. The information from these three sensors is combined to estimate the angular rotation of the sensor [3].

The wearable sensor unit is connected to the computer through Bluetooth connection, following the classic serial port profile of the RN42 wireless module (Microchip Technology Inc). Once the wearable sensor is paired with the computer, the sensor streams orientation data, which can be read through a virtual serial communication port.

### Video Game Description: Active Airlines

The video game in this study has been designed based on the key features identified by previous studies involving the design of serious video games for physical and cognitive rehabilitation or exercise. Previous research has shown that the most relevant key feature is to keep players engaged with a challenge adapted to the skills of a particular user [33-35]. This is a critical point when the users are older adult people, due to their physical and cognitive skills.

Based on the key features described by the literature, we developed the software component of the solution, called Active Airlines. This is a Windows-based application, using the C# language in Unity’s integrated development environment, designed to provide an interactive scenario for assessment and exercise of cervical ROM. The main functionality of the software solution was to control a virtual airplane to reach targets using head motions (Multimedia Appendix 1).

The key factors described in the literature were adapted to fulfill the physical and cognitive skills of our target population, following these criteria:

1. Motor control. The user performs a specific movement that requires the anticipation of feedback; as a result, outcomes may be critical to motor learning. The number of targets, the target size, and the airplane speed are customizable. These targets are shown randomly every time the game starts.
2. Cervical ROM. The distance between targets is customizable as a function of the cervical ROM required to reach them.
3. Cognitive challenge. The video game presents a simple and understandable goal-directed task.
4. Sensitivity to auditory and visual limitations of the target population. There is high contrast between the different elements of the scene and representative sounds when an event occurs.
5. Biofeedback. The airplane moves according to the user’s head movement, and the video game offers visual and auditive stimuli when the user succeeds and fails.
6. Meaningful play. To provide an incentive to keep playing, besides perceiving the immediate result of the reached target via visual and auditive stimuli, a final score of the reached targets is shown.

The control algorithm follows absolute mapping, which means that a given angular orientation of the head always corresponds to the same position of the virtual airplane. Absolute mapping is more interesting than relative mapping (ie, based on a relative variable, such as movement speed or acceleration) for rehabilitation purposes, because the system demands an upright posture to control the game successfully.

The three degrees of freedom of the cervical joint (ie, 3D space), corresponding to flexion-extension, right-left inclination, and right-left rotation, have to be translated into the vertical and horizontal coordinates of the virtual airplane (ie, 2D space). The vertical coordinate (y) is always controlled by the angle of flexion-extension, and the horizontal coordinate (x) can be controlled by the angular inclination or rotation of the head. The software solution integrates a graphical user interface to configure the following options (Multimedia Appendix 2):

1. Control of the horizontal coordinate using inclination or rotational movement.
2. Control of the virtual airplane in 1D (ie, vertical or horizontal axis) or 2D (ie, vertical and horizontal axes).
3. Number of targets to reach.
4. Required cervical ROM to reach the targets (ie, angular sensibility).
5. Level of difficulty (eg, speed of target appearance).

Absolute mapping uses the Euler angles generated from the direction cosine matrix (DCM) using the YZX Euler convention. The x and y coordinates of the airplane were calculated according to the following formulas:




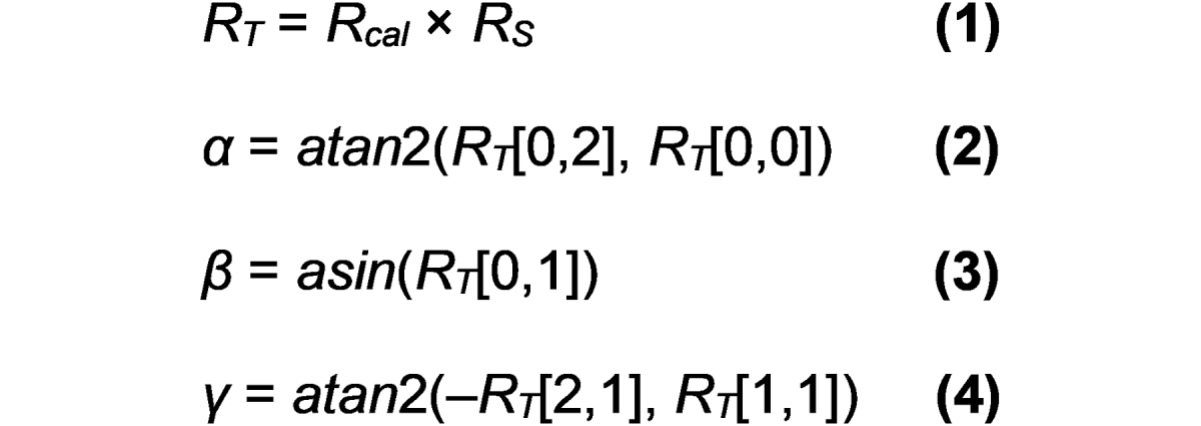




where *R_T_* is the result of multiplying the *R_cal_* matrix (ie, the DCM in neutral posture) by *R_S_* (ie, the DCM in every sample). The x and y values represent the horizontal and vertical coordinates of the airplane (pixels), respectively. *R_H_* and *R_V_* represent the horizontal and vertical distance between targets (pixels), respectively. *α_T_* and *β_T_* represent the required ROM to reach the targets (angular degree).

Before starting the exercise, a calibration process is required to establish the *R_cal_* matrix. The subject maintains a neutral posture (ie, head in the upright position) for 2 seconds, and the physiotherapist calibrates the device by clicking a specific button on the software application.

Finally, the software application includes a database to register the subjects’ data. At the end of the exercise, the application stores the targets reached and missed and the airplane’s trajectory (Multimedia Appendix 3). The number of reached and missed targets together represents the performance score, which is directly related to the motor control of the head.

### Pilot Study Design

The usability analysis and preliminary evaluation of the effects were carried out through a randomized, single-blind, crossover clinical pilot study. Subjects were recruited from the Residencia Municipal de Griñón, a nursing home in Madrid, Spain. The trial was conducted under the criteria of the CONSORT (Consolidated Standards of Reporting Trials) statement [36]. This study was approved by the Ethics Committee of the Centro Superior de Estudios Universitarios La Salle, Universidad Autónoma de Madrid (approval code: CSEULS-PI-181/2017). All participants provided signed informed consent before participation.

As the primary objective of this study was to develop a serious game and then assess its suitability, and not to measure the outcome of the intervention through a randomized controlled trial, the study was not retrospectively registered.

### Participants

Men and women over 65 years of age with chronic neck pain were included. Subjects selected for this study had to meet the following inclusion criteria: be at least 65 years of age; understand, write, and speak Spanish fluently; have suffered from neck pain for more than 12 weeks [37]; scored more than 5 points on the Neck Disability Index (NDI); have no cognitive impairment below 20 points, as assessed by the Mini–Mental State Examination (MMSE) scale; and be willing to undergo treatment. In addition, the following exclusion criteria were used: red-flagged medical history (ie, tumor, fracture, metabolic diseases, and rheumatoid arthritis), fibromyalgia syndrome, previous neck surgery, or neck pain accompanied by vertigo caused by vertebrobasilar insufficiency. The subjects were recruited by word of mouth, through individual interviews with patients.

### Research Team

The research team was composed of three physiotherapists, which included two evaluators and one person who administered the treatment and was in charge of assigning the groups so that the outcome evaluators were blinded to patients’ group assignments. The evaluators were in charge of recording the initial selection data and verifying that participants were eligible to participate in the study. At the end of each treatment block, they collected the data obtained from the participants. All participants were asked not to discuss the treatments they were undergoing with the evaluators so as not to influence the records. In the case of noncompliance, patients were excluded from the trial.

### Randomization: Sequence Generation

All subjects who fulfilled the study selection criteria were randomly assigned to one of two study arm groups (see Interventions section below). Randomization was performed using the online computer program Prism (version 5; GraphPad Software) to assign participants to the serious game group or the conventional exercise group.

### Primary Outcomes

The Suitability Evaluation Questionnaire (SEQ) was designed to test items such as satisfaction, acceptance, and security of use in virtual rehabilitation systems. It is an easy-to-understand questionnaire, with a reasonable number of straightforward and clear questions that are evaluated on a scale from 1 (“not at all” or “very easy”) to 5 (“very much” or “very difficult”). The questionnaire addresses different items related to virtual rehabilitation systems, with 14 questions on feeling, satisfaction, and realism; the last question is open ended, where patients, if they felt uncomfortable, were asked the reasons why. The minimum score is 13 points, and the maximum score is 65 points. Previous research has used the SEQ to evaluate the suitability of virtual rehabilitation in older adults [38].

### Secondary Outcomes

#### Number of Targets Reached in the Serious Game

The number of targets reached by each user in the serious game group was evaluated as a percentage in order to compare the proportion of hits across sessions, representing the performance of the craniocervical motor control. This outcome was only registered during the serious game experimental group sessions; in addition, this outcome was used as complementary data regarding whether the older adults included in the study could improve their game performance over 4 weeks of consecutive sessions. Therefore, this outcome did not allow for any comparison between groups nor for analysis that would evaluate whether improvements in the game scores were associated with changes in pain or disability.

#### Visual Analog Scale

The Visual Analog Scale (VAS) is a horizontal line used to grade the intensity of pain, from no pain to the maximum possible pain. Poor pain control is considered to be above 3 points on the VAS [39], and the minimum detectable change is 2 points [40]. This scale was used to measure neck pain intensity at baseline and at each of the follow-up points of the pilot study by asking each patient to mark a vertical line on the scale representing the intensity of neck pain in the last 24 hours.

### Control Variables

#### Neck Disability Index

The NDI is a self-completed questionnaire with 10 items: intensity of neck pain, self-care, lifting, reading, headache, ability to concentrate, ability to work, ability to drive, sleep activities, and leisure activities. Each of the items has six possible responses representing six progressive levels of functional ability, with scores ranging from 0 to 5. The total score is expressed in percentage terms with respect to the maximum possible score. The completion time is reasonably short, and a validated Spanish version was used [41]. The NDI has been shown to be a responsive scale among older adult patients with nonspecific neck pain [42]. The minimum detectable change is 5 out of 50 points, and a change of 7 points is recommended for achieving a clinically relevant difference [43].

#### Mini–Mental State Examination

To measure cognitive impairment, we used an adapted and validated Spanish version of the Folstein MMSE [44]. This is a screening test for dementia that is also useful in the evolutionary follow-up of dementia. This questionnaire explores short- and long-term memory, orientation, information about everyday events, and calculation capacity. Information was collected by means of an auto-administered questionnaire. The results were evaluated according to the following number of errors: 0 to 2 (normal), 3 to 4 (mild cognitive impairment), 5 to 7 (moderate cognitive impairment), and 8 to 10 (severe cognitive impairment). When using this scale, it is important to consider the educational level of the person taking the test. In cases of low educational level (ie, elementary education), one additional error is allowed for each category. In cases of high educational level (ie, university level), one fewer error is allowed for each category (35 points maximum). Two cutoff points are considered according to age: 24 points for those 65 years of age and older, and 29 points for nongeriatric adults. The classification brackets for points are as follows: 30 to 35 (normal), 24 to 29 (borderline), 19 to 23 (mild), 14 to 18 (moderate), and less than 14 (severe) [44].

### Interventions

#### Overview

For the crossover study, subjects were randomly assigned to one of two study groups. Group A-B started by testing the serious game for 4 weeks (8 sessions), followed by a 4-week washout period, after which they performed conventional exercises for 4 weeks (8 sessions). Group B-A started with the conventional exercises and then tested the game after the washout period. The total study duration was 16 weeks, including a final follow-up measure 4 weeks after the end of all treatments.

#### Serious Game: Treatment A

Treatment A consisted of the use of the Active Airlines serious game twice a week for 4 weeks. The sensor had to be held at forehead level with an elastic band and Velcro, with a Windows computer in front of it in order to run the application correctly; the computer screen was placed at eye level, and the keyboard was placed approximately in a straight line with the xiphoid process. Within the Active Airlines application, parameters could be set, such as difficulty, degrees of rotation, movement to be treated, and number of objects to be picked up. An “easy” difficulty level was selected for all subjects, with a marked maximal mobility of 20º to 30º in order to cover the entire screen (ie, flexion-extension movements moved the plane down or up, respectively, and lateral inclinations moved the plane to the sides), leaving rotation unworked in this study. The location of each of the targets was shown on the screen randomly, and the time elapsed between targets was 5 seconds. The participant performed the exercise twice (ie, two series) in each session, and the application was set up so that the participant aimed to pick up 21 targets per series, for a total of 42 points. Considering that the patient had to perform a combined movement (ie, flexion-extension combined with right-left lateral flexion) to reach each of the targets, the total number of combined movements performed by patients in each session was 42 (ie, one per target), and the total game duration was 210 seconds (ie, 5 seconds per target). At the end of each series, the targets picked up were counted and recorded as a percentage value (ie, the score for the day was recorded as the total percentage from the two series). Once the first series was finished, the exact same procedure was performed again, thus ending the serious game session for that day. In this group, the sessions included only one participant at a time.

#### Conventional Exercise: Treatment B

Treatment B consisted of a therapeutic exercise protocol that was based on two weekly sessions of conventional physical therapy for 4 weeks; this consisted of an exercise program of about 30 to 45 minutes in length for groups of 2 to 4 patients. This program included 5 minutes of stairs, 10 minutes of pedaling, 5 minutes of pulleys, and 5 minutes of obstacle walking. In addition, three sets of 12 repetitions of cervical joint mobility exercises in all ranges were added: cervical flexion-extension, right-left lateral flexion, and cervical rotations. The exercises were not performed with resistance, but were only self-loading at the beginning of the ROM.

### Procedure

Once the informed consent forms were revised and signed by the participants, the VAS, NDI, and MMSE were administered at baseline. The study had a total duration of 16 weeks. First, the therapist assigned each study group to one study arm. One group followed the A-B sequence with an intermediate washout period of 4 weeks. The other group received the treatment in the B-A order, also with a washout period of 4 weeks. Finally, the last assessment of outcomes was carried out 4 weeks after the treatment finished. Thus, the distribution of the evaluators’ measurements was as follows: baseline (0 weeks), after first intervention (4 weeks), washout period (8 weeks), after second intervention (12 weeks), and follow-up period (16 weeks).

In the washout and follow-up weeks, no exhaustive follow-up was performed, and participants were simply reminded, sporadically, to keep up with the previously prescribed exercises. These exercises were based on cervical joint mobility.

The evaluation of the VAS was carried out at all follow-up periods, and the SEQ was included in the evaluation at the end in order to assess the degree of suitability of the inertial sensor and the Active Airlines game.

### Data Analysis

All statistical tests were performed with SPSS Statistics for Windows (version 27; IBM Corp) with a significance level of *P*<.05. Demographic data were analyzed with descriptive statistics and were represented as mean (SD) for each of the variables. The Shapiro-Wilk statistic was used to test the normal distribution of the data. Since this was a randomized crossover clinical trial, it was necessary to analyze the difference in the variables of interest between each of the interventions. For this purpose, the Student *t* test was used for related measures, to compare between groups and within groups.

Also, based on the crossover design, other effects had to be analyzed, such as the residual effect, period effect, and sequence effect. In order to guarantee as high a quality as possible in the analysis, the following tests were performed, according to previous recommendations [45].

To verify that the interventions had an effect over time, the residual effect of the interventions was analyzed by performing a Student *t* test for related samples, comparing the initial measurement with the measurement after the washout period. In the case of statistically significant results, the Wilcoxon signed-rank test was used to repeat the analysis, subdividing by intervention group to identify the intervention that produced the residual effect. The period effect was also analyzed by performing a Student *t* test for related samples, comparing the measures after the first intervention (4 weeks) and after the second intervention (12 weeks).

Finally, to test the sequence effect, the change produced in the variables of interest with each of the interventions was analyzed by comparing the A-B sequence and the B-A sequence. For example, if there was no sequence effect, the value obtained for the VAS variable after having received the serious game intervention should be the same, either in the first period or in the second period. Therefore, the difference between baseline and postintervention for each variable was calculated for each group and compared, based on whether the A-B or B-A sequence was followed.

In addition, for the study of the control variables and variables related to the use of the technology, the Student *t* test for related measures and the repeated-measures analysis of variance (ANOVA) test were used.

## Results

### Overview

**Figure 1:**
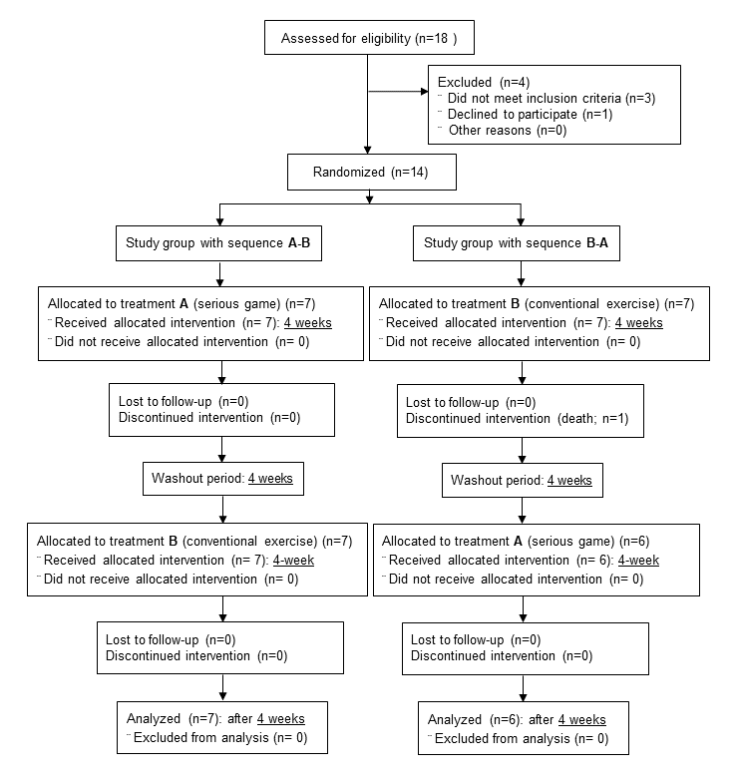
Participant flowchart.

A total of 18 subjects were evaluated for inclusion in the study. Of these, 14 subjects, aged between 71 and 92 years (mean 81.85, SD 6.82), were finally included (Figure 1). There was 1 participant lost due to death in the B-A sequence study group during the first 4-week period of treatment. Demographic variables and all baseline variables showed a normal distribution, with *P* values greater than .05 in the Shapiro-Wilk test. Descriptive data for the demographic variables are shown in Table 1.

**Table 1:** Baseline descriptive data and normality test.

Variable	Value (N=14)	*P* value^a^
Sex (women), n (%)	9 (64)	N/A^b^
Age (years), mean (SD)	81.85 (6.82)	.31
Weight (kg), mean (SD)	68.16 (8.38)	.82
Height (cm), mean (SD)	1.54 (0.07)	.12
BMI, mean (SD)	28.65 (3.58)	>.99
Mini–Mental State Examination score^c^	31.08 (3.01)	.13
Neck Disability Index score^d^	15.77 (8.19)	.98

^a^*P* values are based on the Shapiro-Wilk test, which measures the distribution of variables; variables showed a normal distribution.

^b^N/A: not applicable; the Shapiro-Wilk test cannot be performed on categorical data.

^c^The classification brackets for this scale are as follows: 30 to 35 (normal), 24 to 29 (borderline), 19 to 23 (mild), 14 to 18 (moderate), and less than 14 (severe).

^d^The minimum detectable change for this index is 5 out of 50 points, and a change of 7 points is recommended for achieving a clinically relevant difference.

### Suitability of the Technology

The scores obtained in each of the items of the SEQ are presented in Table 2. The global mean score for the SEQ was 50.38 (SD 5.35) out of 65 points, showing good suitability of the Active Airlines serious game. Most of the patients considered the experience to be very enjoyable and “real” in terms of the virtual environment, and found the information provided to be clear. Also, most of them thought that the game could be very helpful for their rehabilitation.

The results of the SEQ also showed that none of the patients felt any neck pain or discomfort when playing the game, and only 2 patients out of 13 (12%) reported some degree of dizziness, eye discomfort, or disorientation. These two events of dizziness were also recorded separately by researchers as minor adverse effects. Both subjects were able to finish the session and kept participating in the study. No other adverse events occurred during any of the treatment sessions.

Moreover, most patients considered the task to be difficult and the system difficult to use, suggesting that the game presented a challenge for them across the different treatment sessions.

**Table 2:** Suitability of the technology based on results from the Suitability Evaluation Questionnaire (SEQ).

Question	SEQ score, mean (SD)^a^
Q1. How much did you enjoy your experience with the system?	4.92 (0.277)
Q2. How much did you sense being in the environment of the system?	3.92 (1.115)
Q3. How successful were you in the system?	3.85 (1.214)
Q4. To what extent were you able to control the system?	3.62 (1.261)
Q5. How real is the virtual environment of the system?	4.62 (0.961)
Q6. Is the information provided by the system clear?	4.31 (0.947)
Q7. Did you feel discomfort during your experience with the system?	1.00 (0.000)
Q8. Did you experience dizziness or nausea during your practice with the system?	1.54 (1.330)
Q9. Did you experience eye discomfort during your practice with the system?	1.54 (1.330)
Q10. Did you feel confused or disoriented during your experience with the system?	1.23 (0.832)
Q11. Do you think that this system will be helpful for your rehabilitation?	4.69 (0.630)
Q12. Did you find the task difficult?	4.69 (0.855)
Q13. Did you find the devices of the system difficult to use?	4.77 (0.599)
Total for all questions	50.38 (5.35)

^a^Questions were scored on a 5-point Likert scale, ranging from 1 (“not at all”) to 5 (“very much”). Reverse scoring was performed for Q7-Q10, Q12, and Q13, ranging from 1 (“very easy”) to 5 (“very difficult”).

### Number of Targets Reached in the Serious Game

A repeated-measures ANOVA showed that the number of targets reached during serious game–playing increased with each session. The results showed statistically significant effects over time (*F*_1_=22.14; *P*<.01), as the percentage of targets reached during the game progressively increased in each session (Table 3).

**Table 3:** Success in the serious game during each treatment session.

Session	Success (%), mean (SD)^a^
1	68.86 (24.87)
2	71.06 (24.63)
3	82.05 (19.50)
4	82.96 (21.14)
5	85.16 (15.25)
6	83.51 (13.31)
7	86.44 (16.02)
8	89.74 (12.95)
Percentage change from session 1 to 8	24.90 (20.85)

^a^The serious game software only returns the percentage of success in the game.

### Pilot Study Results for Pain

There were no significant differences between the effects of the serious game and conventional exercises when considering all subjects who received each treatment, independent of the study group sequence, but both treatments showed improvements in neck pain intensity (Table 4).

A statistically significant residual effect was found. The Wilcoxon signed-rank test, used as a secondary analysis subdividing by group, showed a statistically significant residual effect only for the conventional exercise intervention (baseline: mean 5.36, SD 1.84; washout: mean 3.21, SD 2.45; Z=–2.38, *P*=.02).

**Table 4:** Comparison between treatments, intratreatment changes, and residual effect.

Treatment^a^	Baseline VAS^b^ score, mean (SD)	Posttreatment VAS score, mean (SD)	*P* value^c^	Washout period VAS score (residual effect), mean (SD)	*P* value^c^
Serious game	4.92 (1.88)	3.77 (1.92)	<.001	3.69 (2.13)	.01
Conventional exercise	4.92 (1.88)	3.46 (2.22)	<.001	3.69 (2.13)	.01

^a^No statistically significant differences between treatments were detected in the measurement after the intervention (serious game vs conventional exercise).

^b^VAS: Visual Analog Scale.

^c^*P* values are based on the Student *t* test.

Period effect analysis revealed that there were statistically significant differences between the end of the first period and the end of the second period. The mean VAS score at baseline was 4.92 (SD 1.88), the score after the first treatment was 4.15 (SD 1.57), and the score after the second treatment was 3.08 (SD 2.36; *P*=.003).

Finally, the sequence effect analysis for the serious game intervention showed that pain was reduced to a greater extent with the B-A sequence (VAS score mean difference –1.64, SD 0.75) than with the A-B sequence (VAS score mean difference –0.58, SD 0.49). On the other hand, for the conventional exercise treatment, pain was reduced to a greater extent with the A-B sequence (VAS score mean difference –2.08, SD 1.02) than with the B-A sequence (VAS score mean difference –0.93, SD 0.45). Table 5 shows the changes in pain that occurred after treatment in each of the study group sequences.

**Table 5:** Sequence effect analysis.

Treatment and sequence^a^			VAS^b^ score, mean difference (SD)^c^		*P* value^d^
**Serious game**					
	A-B	–0.58 (0.49)		.01	
	B-A	–1.64 (0.75)			
**Conventional exercise**					
	A-B	–2.08 (1.02)		.04	
	B-A	–0.93 (0.45)			

^a^Sequence A-B is serious game followed by conventional exercise; sequence B-A is conventional exercise followed by serious game.

^b^VAS: Visual Analog Scale.

^c^This value represents the mean difference between baseline and posttreatment measures.

^d^*P* values are based on the Student *t* test. *P* values for each group are reported in the top row of that group.

## Discussion

### Principal Findings

This study allowed for the development of a serious game to provide a suitable solution for the performance of task-oriented cervical exercises for people with neck pain.

The older adult population included in the study showed very good results in terms of satisfaction, acceptance, and security when using this technology. Moreover, minor adverse events were scarce in the pilot population sample included in the study. Therefore, the older adult population aged over 80 years with chronic pain might benefit from this intervention, but some factors would need further research, such as technological acceptance, visual and hearing disorders, and cognitive impairments, among others, which can become barriers for the success of the intervention.

Although the design of the study did not allow for measuring treatment adherence, the playful approach and integrated technology used may be capable of increasing adherence to the exercise treatment. Further research is needed to investigate whether these technologies are associated with higher adherence or patient motivation compared with conventional exercise programs, which can sometimes be considered more repetitive or boring [12].

The main clinical implication from this study is that patients with neck pain could safely use this serious game with high levels of satisfaction and acceptance. Although the clinical findings from the pilot study are limited and do not allow for medium- or long-term evaluation of its effects, it can be hypothesized that patients’ satisfaction and adherence to exercise may be increased when performing therapeutic exercise through the serious game. Moreover, this serious game has the potential to be used in a telerehabilitation context by physiotherapists; this could result in important advantages regarding cost-effectiveness [46] and the possibility to perform therapeutic exercise at home, without having to make tiring journeys [47], which could be especially relevant at some stages of physiotherapy treatment in the older adult population with neck pain.

The main clinical findings of the pilot study are as follows: (1) both conventional exercise and the use of the serious game had the same effect in reducing neck pain in the older adult population and (2) the A-B sequence (ie, playing the serious game first followed by conventional exercise) reduced pain more than the B-A sequence (ie, conventional exercise first followed by playing the serious game).

On the one hand, the results of this study appear to support the findings of another recent study that suggested that performing exercises with immersive VR is not superior to exercises alone without VR among young adult patients with neck pain [48]. On the other hand, the results of this research are not supported by those found in another study that compared nonimmersive VR exercises with proprioceptive training in patients with neck pain, using eight sessions over a period of 4 weeks. That study observed that patients in the VR group improved more in terms of pain and disability than the group that performed proprioceptive exercises [49].

Another study in which VR was added to neck exercises in one group and compared to a group that performed exercises alone during four to six treatment sessions found that only the group that included VR improved more in terms of disability and ROM in rotation. However, there was no improvement over the exercise-only group in terms of pain intensity. Those results support the ones obtained in this study; although we did not measure the ROM variable, patients improved in accuracy when playing the video game in successive sessions [18].

The results of this study were novel in terms of the use of a serious game in a population of adults over 70 years of age, but we must analyze a series of important limitations for their possible applicability in clinical practice. First, the characteristics of the pilot study with a reduced sample size limit the generalizability of the results in terms of the suitability of the serious game in this older adult population and its effects on the treatment of neck pain. Second, the study had a crossover versus parallel design; therefore, it is more difficult to demonstrate the isolated effects of each therapy. Third, a control group was not included to investigate whether the therapies used had a greater effect than the natural evolution of neck pain. Fourth, psychological variables such as kinesiophobia or anxiety, which have been shown to influence the effects of interventions for neck pain in other studies with VR, were not measured. The fifth limitation is that the washout period was shown to be ineffective according to the statistical analysis of this study; for future studies, washout periods longer than 4 weeks should be considered.

### Conclusions

The serious game developed in this study showed good suitability when used in a population of adults over 70 years of age with chronic neck pain. It was a safe method for performing task-oriented cervical exercises, and patients reported very good levels of satisfaction and acceptance after the use of this technology. Although preliminary results on the effects of using the serious game showed short-term improvements in pain intensity, further research with larger samples is needed.

## Appendix

Multimedia Appendix 1 Screenshot of the Active Airlines serious game.

Multimedia Appendix 2 Settings for the graphical user interface of the Active Airlines serious game.

Multimedia Appendix 3 Subjects' data regarding targets reached and missed in the Active Airlines serious game.

## Abbreviations

ANOVA: analysis of variance
CONSORT: Consolidated Standards of Reporting Trials
DCM: direction cosine matrix
MMSE: Mini–Mental State Examination
NDI: Neck Disability Index
ROM: range of motion
SEQ: Suitability Evaluation Questionnaire
VAS: Visual Analog Scale
VR: virtual reality
