# Supplementary figures and images for: A Serious Game for Performing Task-Oriented Cervical Exercises Among Older Adult Patients With Chronic Neck Pain: Development, Suitability, and Crossover Pilot Study
Source: JMIR Serious Games. 2022 Feb 1;10(1):e31404. doi: 10.2196/31404 (PMC8848226; doi:10.2196/31404)

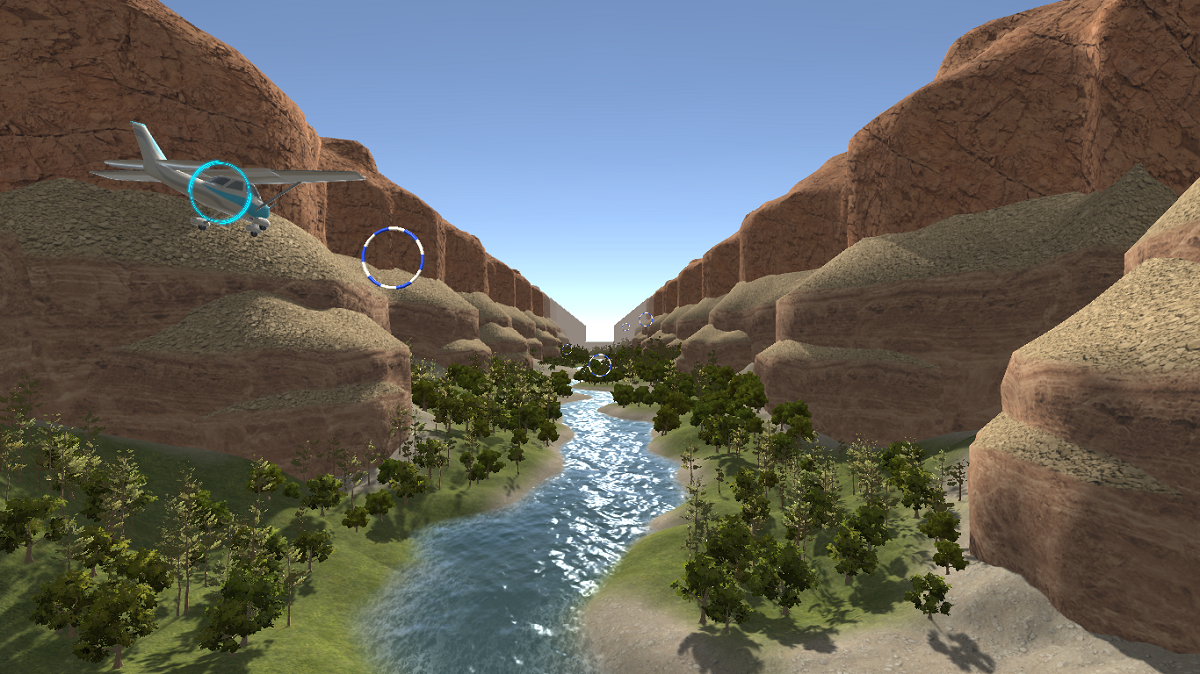

Supplement: Multimedia Appendix 1 [file games_v10i1e31404_app1.png]

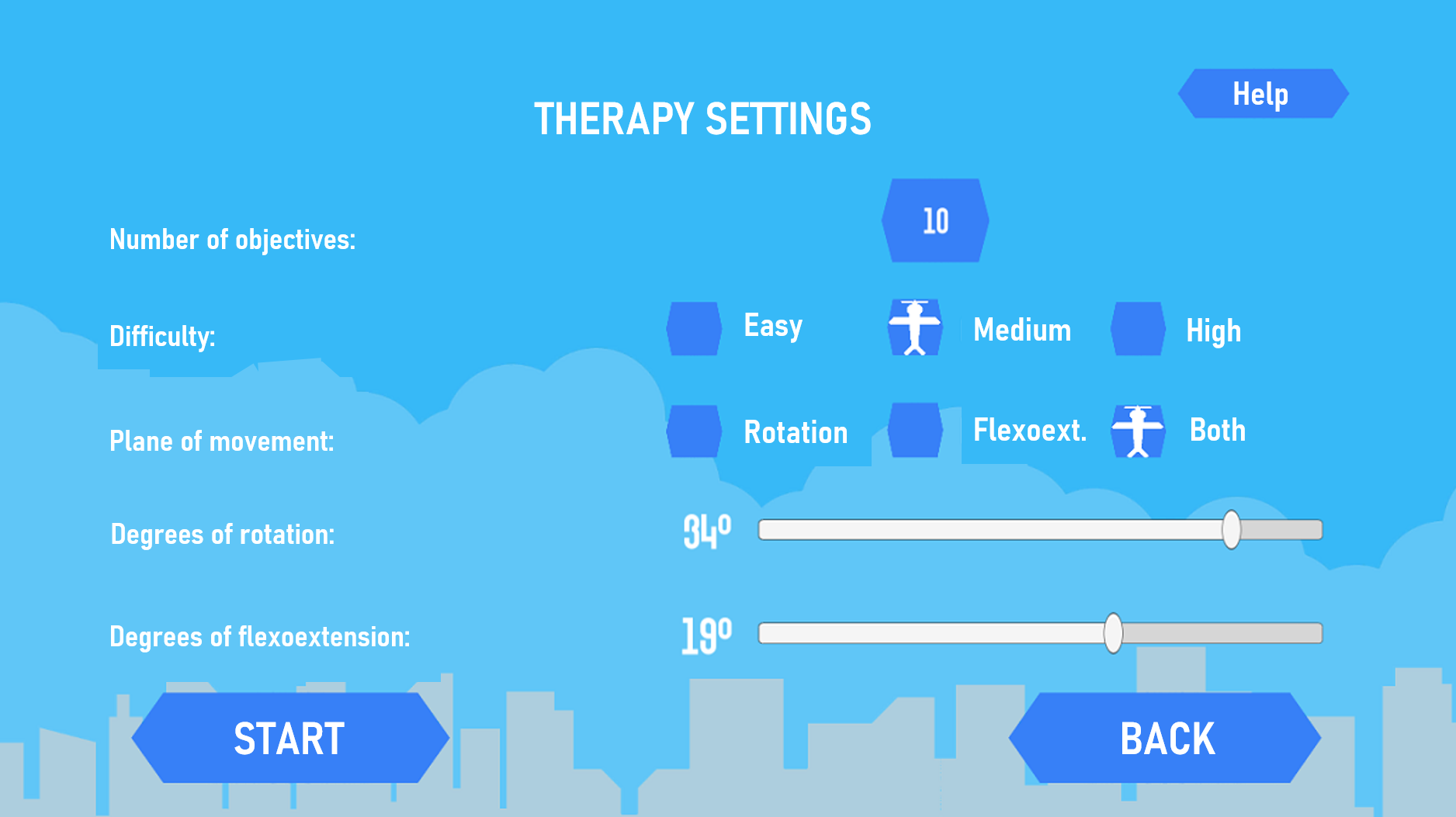

Supplement: Multimedia Appendix 2 [file games_v10i1e31404_app2.png]

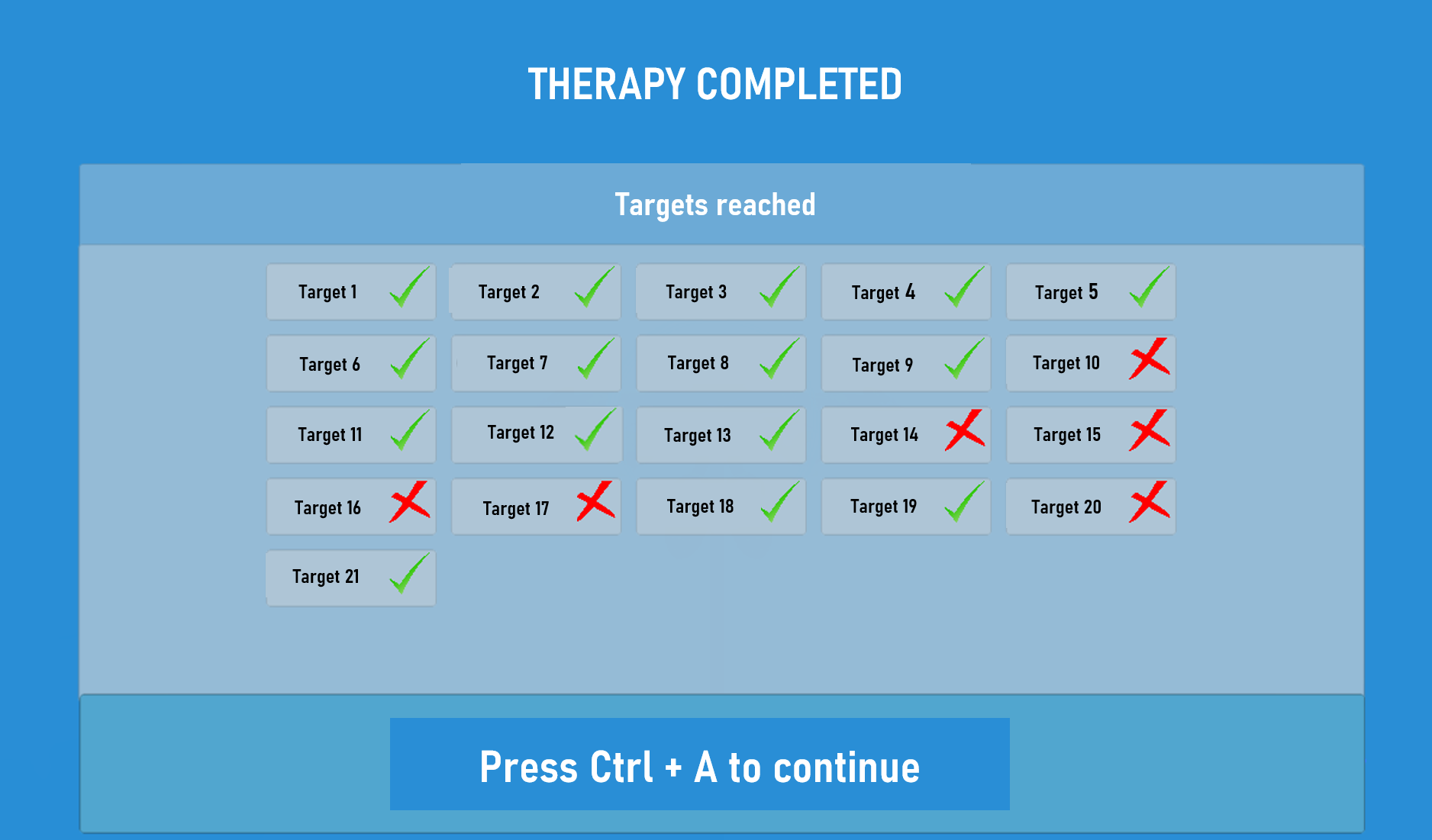

Supplement: Multimedia Appendix 3 [file games_v10i1e31404_app3.png]
